# A dietary perspective of cat-human interactions in two medieval harbors in Iran and Oman revealed through stable isotope analysis

**DOI:** 10.1038/s41598-023-39417-7

**Published:** 2023-07-29

**Authors:** Anastasia Brozou, Benjamin T. Fuller, Bea De Cupere, Anaïs Marrast, Hervé Monchot, Joris Peters, Katrien Van de Vijver, Olivier Lambert, Marcello A. Mannino, Claudio Ottoni, Wim Van Neer

**Affiliations:** 1 Department of Biology, University of Rome “Tor Vergata”, Via della Ricerca Scientifica 1, 00133 Rome, Italy; 2 Géosciences Environnement Toulouse, UMR 5563, CNRS, Observatoire Midi-Pyrénées 14, 31400 Toulouse, France; 3 Operational Directorate Earth and History of Life, Royal Belgian Institute of Natural Sciences, Vautierstraat 29, 1000 Brussels, Belgium; 4 UMR AASPE 7209, Muséum National d’Histoire Naturelle, CP 56, 43 Rue Buffon, 75005 Paris, France; 5 CNRS UMR 8167, Orient & Méditerranée, 27 Rue Paul Bert, 94200 Ivry-Sur-Seine, France; 6 ArchaeoBioCenter and Institute of Palaeoanatomy, Domestication Research, and the History of Veterinary Medicine, Ludwig Maximilian University Munich, Kaulbachstr. 37, 80539 Munich, Germany; 7 SNSB, State Collection of Palaeoanatomy Munich, Karolinenplatz 2a, 80333 Munich, Germany; 8 Department of Archaeology and Heritage Studies, Aarhus University, Moesgård Allé 20, 8270 Højbjerg, Denmark

**Keywords:** Animal behaviour, Stable isotope analysis

## Abstract

Cats are hypercarnivorous, opportunistic animals that have adjusted to anthropogenic environments since the Neolithic period. Through humans, either by direct feeding and/or scavenging on food scraps, the diet of cats has been enriched with animals that they cannot kill themselves (e.g., large mammals, fish). Here, we conducted carbon and nitrogen stable isotope ratio analysis to reconstruct the diet of medieval cats and investigate cat-human interactions in two medieval harbor sites (Qalhât, Oman and Siraf, Iran). The analysis included 28 cat individuals and 100 associated marine and terrestrial faunal samples pertaining to > 30 taxa. The isotopic results indicate a high marine protein-based diet for the cats from Qalhât and a mixed marine-terrestrial (C_4_) diet for the cats from Siraf. Cats at these sites most likely scavenged on both human food scraps and refuse related to fishing activities, with differences in the two sites most likely associated with the availability of marine resources and/or the living conditions of the cats. By shedding light on the dietary habits of cats from two medieval harbors in the Arabian Gulf and Gulf of Oman, this study illustrates the potential of stable isotope analysis in reconstructing human-cat interactions in the past.

## Introduction

Cats are hypercarnivorous animals, requiring a threefold consumption of protein compared to omnivorous species^1^. Their high protein requirements stem from their metabolic adaptation to use protein and fat as energy sources^2,3^, as well as from their increased need for certain amino acids, such as taurine and arginine, which they cannot synthesize themselves^4–6^. With a preference for consuming multiple, small meals throughout the day^7^, cats prey on small mammals, birds, reptiles, amphibians and invertebrates^8–10^, with prey size decreasing with increasing hunger^11^. Predation occurs also as part of teaching or playing and, thus, not always results in the consumption of the prey^12^. However, both the predation rate and the prey diversity seem to be higher in rural areas, where anthropogenic food is scarcer^13^. Being opportunistic hunters with an ability to adjust rapidly to changing environments^14^, wild cats seem to have exploited the new hunting grounds that emerged from the development of permanent settlements, following the onset of agricultural activities^15,16^. This resulted in their adaptation to human presence, and later on to the consumption of foods facilitated by humans^17^. Either from direct feeding and/or from scavenging on human food scraps, cats have acquired access to animal taxa that they are not able to kill themselves, such as large mammals and fish^18,19^.

Today, the pet food industry incorporates a wide range of ingredients, including even foods of plant origin, such as grains and vegetables^18,19^. Unlike dogs^20^, a cat’s digestive system is not adapted to starch-rich foods; however, milled and cooked plant material can be metabolized^7^, and constitute part of a healthy diet, provided that this is nutritionally complete and balanced^21^. Fish is an important component of pet food, with cats consuming an estimated 6% of all wild caught fish^22^. In nature, however, both domestic and wild cats rarely capture fish. The bulk of evidence on their dietary preferences across four continents shows a very low predation rate (0.3%) of fish for wild, feral and domestic cats^23,24^. Although actual, active capturing of fish by cats seems limited, they feed on fish when access is facilitated^23^. Thus, fish is more often consumed by domestic rather than wild cats^23^. A recent isotopic study by Krajcarz et al.^17^, for example, revealed the consumption of marine protein by domestic cats populating medieval harbor towns in northern Europe.

Currently, information on the paleodiet of cats is scarce, possibly due to the fact that their bones are not often recovered in human food refuse or other anthropogenic contexts. The rarity of cat remains may also be a reason why its domestication history has been, so far, minimally investigated^25–28^. Cat remains are most often isotopically analyzed as single specimens, being part of local isotopic baselines (e.g., Refs.^29–33^). Recent studies, however, focused on the paleodietary reconstruction of domestic and wild cats by analyzing tens of individuals^16,17^. Krajcarz et al.^16^ suggested that the ancestor of the domestic cat, the Near Eastern wildcat *Felis silvestris lybica*, lived in Poland as a free-roaming cat, feeding on synanthropic pests, in close association with Early Neolithic farming communities. In another study, Krajcarz et al.^17^ revealed the influence of regional factors (i.e., socio-economic and geomorphological) on cat dietary patterns in medieval northern Europe.

Dietary reconstructions of ancient cats can, thus, provide information on regional food availabilities (i.e., how people adapted to local environments) and, ultimately on human-cat interactions. Aiming at reconstructing the dietary patterns of cats from two medieval harbors in the Arabian Gulf and the Gulf of Oman, in the present study we conducted carbon and nitrogen stable isotope ratio analysis on 47 cat samples and more than 200 associated faunal samples. By developing local baselines and by focusing on two sites with tens of cat remains as well as with historical and archaeological evidence for a long tradition in seafaring and sea fishing^34–41^, this study seeks to shed light on fish consumption by cats in the past and on medieval human-cat interactions in the Arabian Gulf and Gulf of Oman.

## Materials and methods

**Figure 1:**
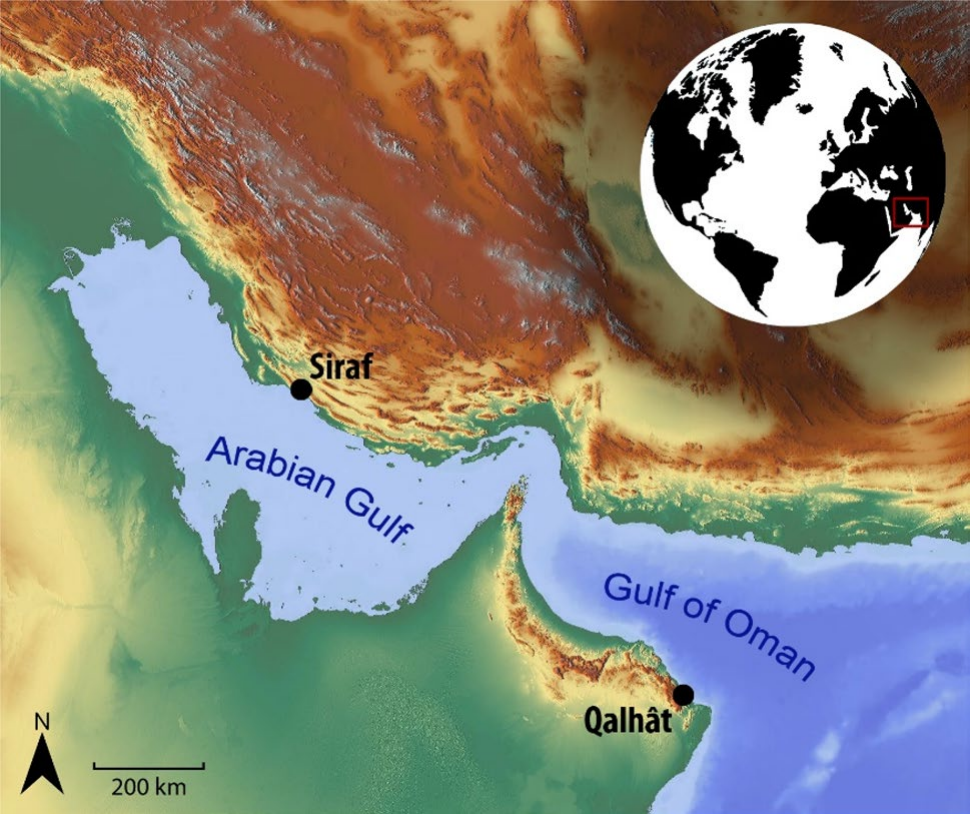
Location of Siraf and Qalhât. Background relief map: released under Creative Commons, CC0 (https://maps-for-free.com), no alteration applied. Miniature globe: released under Creative Commons, CC BY-SA 3.0 (Globe_terrestre_Orange_te_Bleu.svg; Commons Wikimedia). The originally orange-colored continental landmasses and the blue sea surfaces (author: Mouh2jijel) have been converted to a black and white version, respectively, using Photoshop CS 5.5. North arrow, scale and text labels were created using Photoshop CS 5.5.

The cat remains and associated fauna analyzed in this study are from two medieval harbor towns, Siraf in Iran and Qalhât in Oman (Fig. 1). The faunal spectra of the two sites can be adequately compared since in both cases, the zooarchaeological analyses were carried out on large animal refuse accumulations that were sampled in the same way (i.e., by hand-collection). Table 1 presents the relative importance of the major food animals at Qalhât and Siraf, based on the zooarchaeological evidence.

**Table 1:** Proportions (%) at Siraf and Qalhât of (1) the major animal groups, (2) the main domestic species, and (3) the major ecological fish groups, based on identifications provided in von den Driesch and Dockner^36^ for Siraf and in Marrast^37^ and Monchot^46^ for Qalhât. The number of specimens on which the percentages were calculated are given in brackets. For Siraf, the numbers correspond to specimens that date to the first millennium CE, which is the period from which almost all samples for isotopic analysis originate.

Major animal groups	Siraf (n = 14,952)	Qalhât (n = 8945)
Fish	33.9	53.1
Domestic animals	65.3	46.8
Wild birds	0.23	0.07
Terrestrial wild mammals	0.05	0.04
Marine mammals	0.09	0.02
Marine turtles	0.43	–

Domestic species	Siraf (n = 9768)	Qalhât (n = 4184)
Sheep and goat	93.8	95.1
Cattle	3.0	0.6
Camel	0.3	–
Pig	1.6	–
Chicken	1.3	4.3

Major ecological fish groups	Siraf (n = 5063)	Qalhât (n = 4749)
Scombrids	20.1	53.7
Carangids	17.4	20.1
Pelagic sharks	0.4	5.0
Other pelagic fish	–	4.6
Total pelagic	37.8	83.4
Sparids	28.7	5.1
Other coastal fish	33.5	11.5
Total coastal	62.2	16.6

Qalhât, located near the easternmost tip of the Arabian Peninsula, was a large town occupied between the eleventh and sixteenth centuries CE. It was one of the major ports of the region between the thirteenth and fifteenth centuries, when it dominated the maritime trade in the western Indian Ocean and the Arabian Gulf. It was struck by an earthquake at the end of the fifteenth century, then plundered by the Portuguese in 1508 and finally completely abandoned in the second half of the sixteenth century^42^. Fieldwork since 2008 has revealed the layout of medieval Qalhât and has located the town’s major buildings. The cat remains included in the present study belong to 12 domestic cats (of which 3 were non-adult, weaned individuals, cf. Supplementary Table 1) and date between the fourteenth and sixteenth centuries CE. They were found in a cistern (B2), the Great Mosque (B12) and a large domestic building (B94). The remaining species come from the domestic building B94 (with the exception of a finless porpoise and a red fox, which were found in buildings B13 and B95, respectively) and include 54 fish belonging to 15 taxa (cf. Supplementary Table 3), 24 domestic animals (sheep, goat, chicken, dog and donkey), 5 wild birds (4 taxa), 9 wild terrestrial mammals (gazelle, red fox, hare and rat) and 3 delphinoids (cf. Supplementary Table 2). The cat remains from medieval Qalhât were previously described from a zooarchaeological^43,44^ and palaeopathological point of view^45^, while the remaining species are discussed elsewhere by Marrast (fish^37^) and Monchot (other vertebrates^46^).

Siraf is located in the Arabian Gulf and was an important harbor since Sassanid times (between 300 and 600 CE) and in particular between 800 and 1050 CE, when it was a transshipment port for goods coming from India, the Far East and Eastern Africa^47^. Siraf was a thriving, rich town until the end of the tenth century CE, when the area was struck by an earthquake. Afterwards, parts of the town were rebuilt and remained partly inhabited until the sixteenth century CE. An estimated 200,000 faunal remains were recovered from the excavations carried out between 1966 and 1973, of which about 10% was analyzed^36^. The studied material is from the area of the Great Mosque (site B) and consists mainly of remains collected from the shops that surrounded the mosque on three sides. About 80% of the faunal remains date to the most flourishing periods of the town, between 300 and 1050 CE, while the remaining 20% date to later periods (1050 until the early 16th c. CE). The material selected for the present study comes mainly from first millennium CE contexts (chronological units 1a and 1b^36^). This includes 35 domestic cats (of which 2 were non-adult, weaned individuals, cf. Supplementary Table 1), 51 fish belonging to 15 taxa (cf. Supplementary Table 3), 7 sea turtles, 45 domestic animals (sheep, goat, cattle, pig, chicken and dog) and 6 rats (cf. Supplementary Table 2).

Collagen was extracted at the Moesgaard Archaeo-Science Laboratory at Aarhus University (MOS; Denmark) and at the Royal Institute for Cultural Heritage (RICH; Belgium). More specifically, at the Moesgaard Archaeo-Science Laboratory, whole bone pieces were demineralized in 0.5M or 0.25M HCl (depending on preservation) at 4 °C, gelatinized in 0.01M HCl (pH adjusted to ~ 3) at 65 °C for ~ 48 h, purified using an EZEE filter, frozen at − 30 °C and freeze-dried for 48 h. Collagen from bone samples with poor quality indicators was re-extracted, when bone was available, and further purified using pre-cleaned 30 kDa Amicon Ultra Centrifugal Filters (indicated in Supplementary Table 1). In one instance (QALrat04), leftover collagen was ultra-filtered and re-analyzed. Collagen from fish bone samples with poor quality indicators was re-extracted, when bone was available, and treated with 0.1M NaOH prior to gelatinization (indicated in Supplementary Table 3), with the aim to remove humic substances^48^. Collagen extraction at the Royal Institute for Cultural Heritage is described in detail by Wojcieszak et al.^49^. Several studies (e.g., Refs.^50–53^) have demonstrated that stable isotopic values, and thus paleodietary interpretations, are not influenced by the use of different collagen extraction procedures.

The carbon and nitrogen stable isotope ratios of the samples were determined using a Thermo Flash HT/EA elemental analyzer linked to a Thermo Delta V Advantage isotope ratio mass spectrometer (IRMS) via a ConfloIV interface (Thermo Scientific) at the Department of Earth and Environmental Sciences of KU Leuven (Belgium). Data calibrations were done using one international (IAEA-600) and two in-house standards (Leucine and muscle tissue of Pacific Tuna) that were prior calibrated against certified standards. The international and in-house standards were measured at regular intervals throughout each analytical run, and their standard deviations defined the analytical error to be better than 0.11‰ for δ^13^C and 0.08‰ for δ^15^N. The isotopic results are presented as the ratio of the heavier to the lighter isotope (^13^C/^12^C, ^15^N/^14^N) and are reported relatively to internationally defined standards (VPDB, AIR) as *δ* values in units per mil (‰).

For estimating the relative contribution of potential food sources to the diet of the cats, we used the Bayesian mixing model FRUITS (Food Reconstruction Using Isotopic Transferred Signals; version 3.0)^54^. The input parameters used for the mixing model are reported in Supplementary Table S5. Wilcoxon signed rank test was performed using the software R (v. 4.1.3.)^55,56^ to statistically compare the isotope values of the cats from the two sites. A parametric test was conducted as the size of group samples was small (n < 20)^57^.

## Results

### Collagen preservation

**Figure 2:**
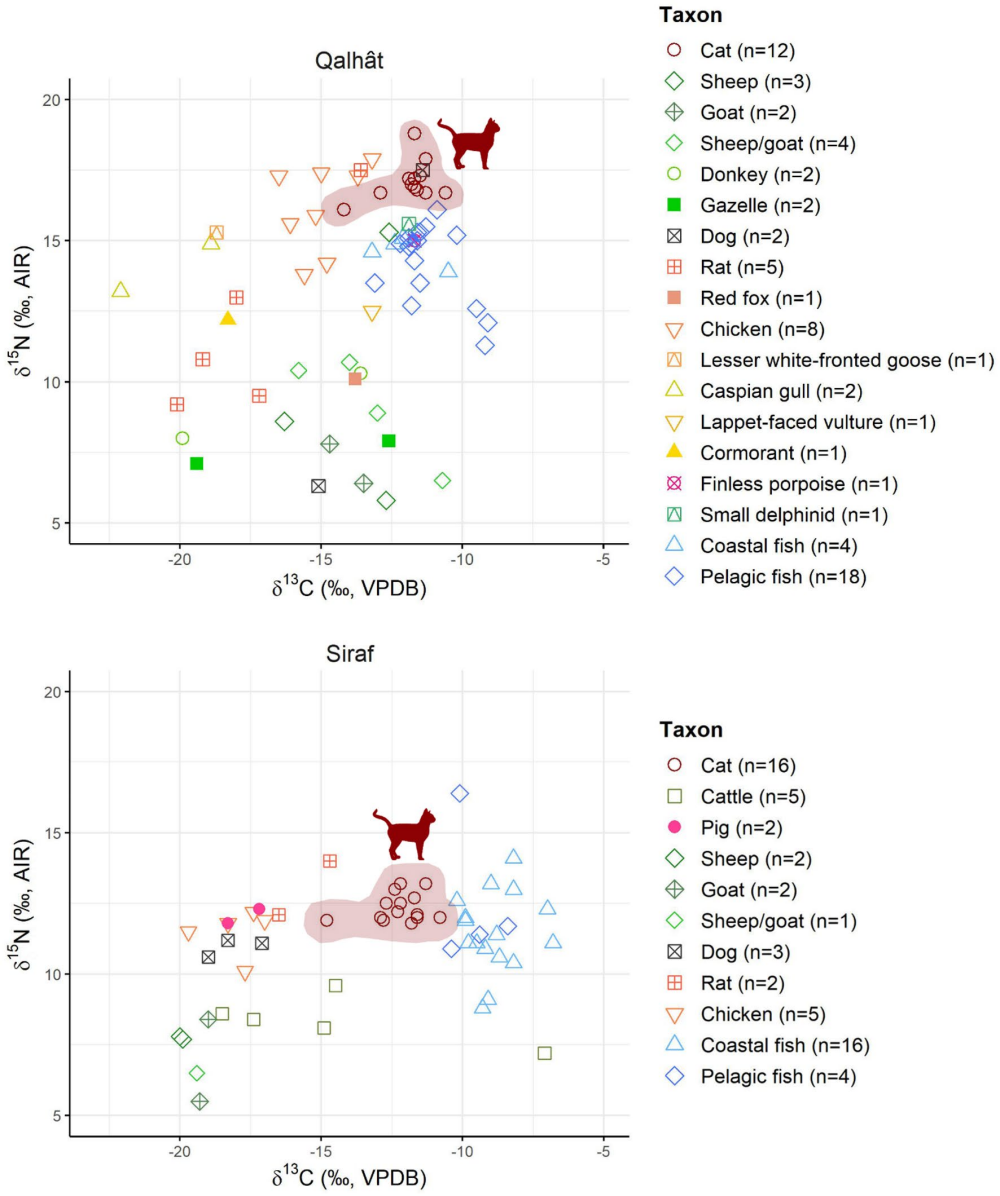
δ^13^C and δ^15^N results of the cats and other faunal remains from Qalhât and Siraf. Supplementary Table S3 presents a list of the coastal and pelagic fish from the two sites.

**Figure 3:**
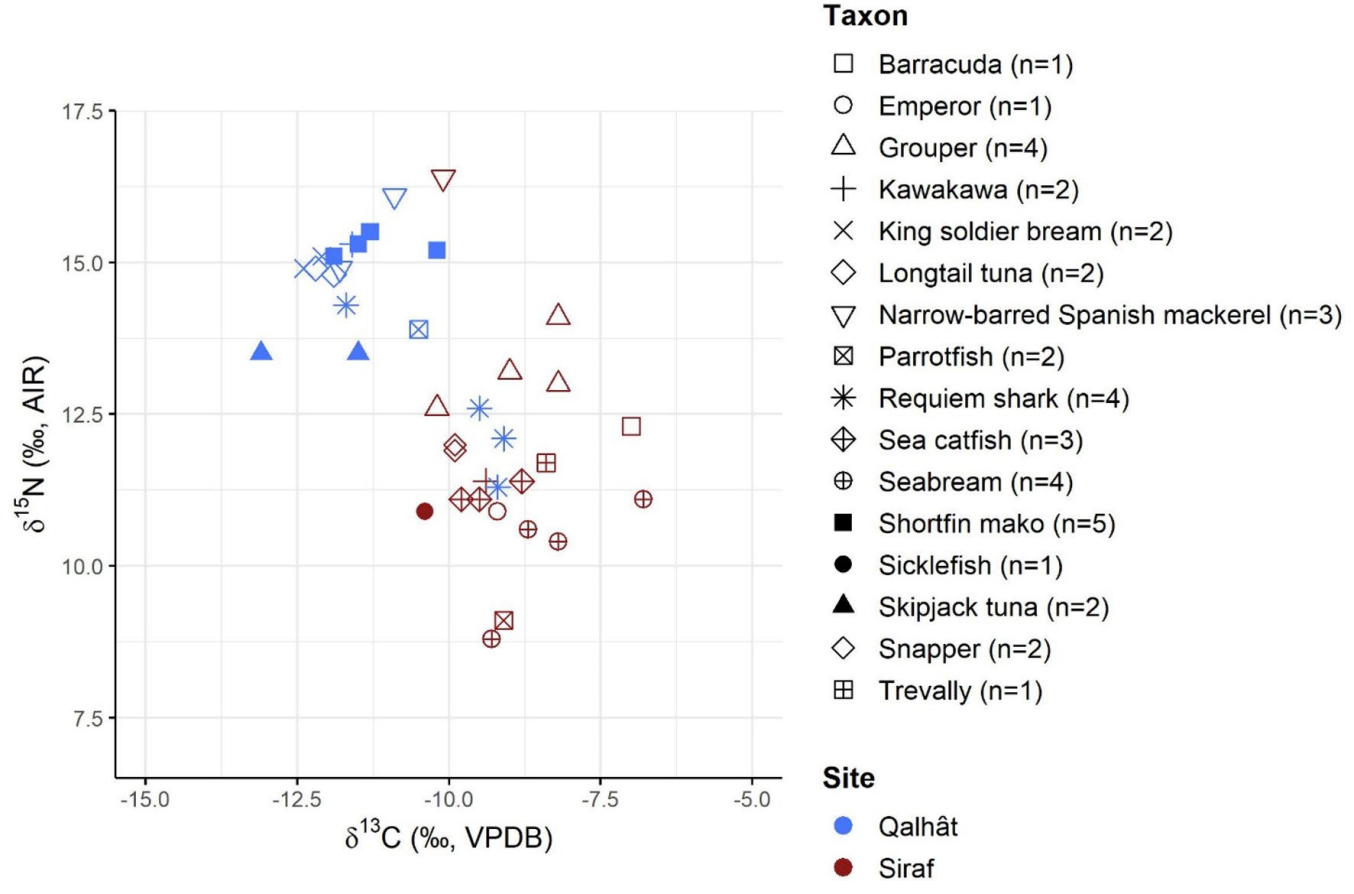
δ^13^C and δ^15^N results of the different fish taxa from Qalhât and Siraf.

All 12 cat samples from Qalhât and 10 out of 35 cats from Siraf yielded good quality collagen (i.e., > 13% for %C, > 4.8% for %N, 2.9–3.6 for C:N and ≥ 1% for collagen yield^58–60^). Another six cats from Siraf (cf. Supplementary Table 1) showed low collagen yields (< 1%), but acceptable %C, %N and C:N. Thus, their values were used for interpretation. Moreover, a total of 42 fish samples, 22 from Qalhât and 20 from Siraf, yielded collagen within the acceptable ranges for %C, %N and C:N, with 22 of them having a collagen yield < 1% (cf. Supplementary Table 3). From the remaining fauna, a total of 58 samples, 36 from Qalhât and 22 from Siraf, yielded acceptable collagen, with one sample from Siraf (SRrat02) having a low yield (cf. Supplementary Table 2). The isotopic values of all the faunal remains from the two sites, which are interpreted, are shown in Figs. 2 and 3.

### Isotopic results from Qalhât

The cats from Qalhât have a mean δ^13^C value of − 11.8‰ (± 0.9‰) and a mean δ^15^N value of 17.1‰ (± 0.7‰). These isotopic values are almost one trophic level above the values of the fish from this site^61,62^, indicating that cats had a diet highly dependent on marine protein. This is further supported by the results of the mixing model (Supplementary Table S6), which suggest that 41 ± 21% of the protein consumed by cats was marine. The fish that yielded acceptable isotopic results have a mean ± SD δ^13^C value of − 11.4 ± 1.1‰ and a mean ± SD δ^15^N value of 14.4 ± 1.2‰ (cf. Supplementary Table 3). Two marine mammals (finless porpoise and small delphinid) have δ^13^C values of − 11.7‰ and − 11.9‰ and δ^15^N values of 15.0‰ and 15.6‰, respectively, reflecting the small fish, crustaceans, cephalopods and/or other mollusks that these two taxa consume. One rat (QALrat01) and one dog (QALdog01) have values that are similar to the values of the cats (i.e., δ^13^C: − 13.6‰ and − 11.4‰, respectively, and δ^15^N: 17.5‰), indicating a significant contribution of marine protein in their diet as well. The remaining four rats have isotope values (δ^13^C mean ± SD: − 18.6 ± 1.3‰ and δ^15^N mean ± SD: 10.6 ± 1.7‰) that reveal different amounts of C_3_, C_4_ and marine protein in their diet, while the second dog (QALdog02) has one of the lowest δ^15^N values reported in this study (6.3‰), which could indicate a diet based primarily on C_3_ and C_4_ plants (δ^13^C: − 15.1‰)^20,63–65^.

With a mean ± SD δ^13^C value of − 15.0 ± 1.1‰ and a mean ± SD δ^15^N value of 16.2 ± 1.5‰, the isotope values of the eight chickens reveal differential amounts of marine protein in their diet. Two wild bird species have δ^13^C (Caspian gull: − 18.9‰ and − 22.1‰ and lappet-faced vulture: − 13.2‰) and δ^15^N (Caspian gull: 14.9‰ and 13.2‰ and lappet-faced vulture: 12.5‰) values that reflect their scavenging nature and the consumption of both terrestrial and marine protein. One lesser white-fronted goose has a δ^13^C value of − 18.7‰ and a δ^15^N value of 15.3‰, which perhaps reflect the consumption of coastal plants (including halophytes^66^) that were affected by sea-sprays and soil salinity^67,68^, while one cormorant with a δ^13^C value of − 18.3‰ and a δ^15^N value of 12.2‰ likely fed on brackish or a mix of freshwater fish and amphibians as well as marine fish. The latter species may have foraged in Khors (i.e., the typical estuarine habitats found along the Arabian Peninsula’s coast)^69,70^. From the wild terrestrial mammals, two gazelle specimens with similar δ^15^N values (7.1‰ and 7.9‰) and a 6.8‰ difference in δ^13^C values (− 19.4‰ and − 12.6‰) reflect the consumption of plants that follow different photosynthetic pathways (i.e., C_3_ and C_4_, respectively), while one red fox has a δ^13^C value of − 13.8‰ and a δ^15^N value of 10.1‰, which indicate the consumption of small, wild mammals that were highly dependent on C_4_ plants. From the domestic mammals, sheep and goats have similar isotope values that range between − 10.7‰ and − 16.3‰ for carbon and between 5.8‰ and 10.7‰ for nitrogen, reflecting mainly the consumption of C_4_ plants, partially at coastal sites^71^. Apart from a high variety of wild C_4_ plants in the area^66^, archaeobotanical evidence from the region reveals the presence of cultivated C_4_ plants, such as millet^72,73^ and sorghum^74^, while historical sources attest that cultivated plants were intended for both human and animal consumption^75^. One sheep has a δ^13^C value of − 12.6‰ and a δ^15^N value of 15.3‰, reflecting the consumption of marine fish, which, based on medieval written testimonies, were used as fodder in the area during the sampled period^35^. Finally, two donkeys have a δ^13^C value difference of 6.3‰ (− 19.9‰ and − 13.6‰) and a δ^15^N value difference of 2.3‰ (8.0‰ and 10.3‰) that reflect a dependence on C_3_ and C_4_ plants, respectively, the latter perhaps from coastal environments.

### Isotopic results from Siraf

Cats from Siraf have a mean ± SD δ^13^C value of − 12.1 ± 0.9‰ and a mean ± SD δ^15^N value of 12.3 ± 0.5‰, suggesting the consumption of protein from C_4_ sources and perhaps some contribution of marine protein. The lesser contribution of marine protein to the diet of these cats is also supported by the results of the mixing model (Supplementary Table S6), which suggest that 34 ± 19% of the protein consumed by the cats was derived from fish and 37 ± 21% from herbivorous mammals. The 20 fish specimens that yielded acceptable isotopic results have a mean ± SD δ^13^C value of − 9.8 ± 4.0‰ and a mean ± SD δ^15^N value of 12.0 ± 2.2‰ (cf. Supplementary Table 3). From the terrestrial animals, three dogs (δ^13^C mean ± SD: − 18.2 ± 0.9‰, δ^15^N mean ± SD: 11.0 ± 0.3‰), five chickens (δ^13^C mean ± SD: − 18.0 ± 1.0‰, δ^15^N mean ± SD: 11.5 ± 0.8‰) and two pigs (δ^13^C: − 18.3‰ and − 17.2‰, δ^15^N: 11.8‰ and 12.3‰) have similar isotope values that indicate a primarily C_3_-based diet with some contribution of protein from C_4_ and/or marine sources. Two rats have δ^13^C values of − 14.7‰ and − 16.5‰ and δ^15^N values of 14.0‰ and 12.1‰, respectively, indicating differential access to marine protein. For the domestic mammals, sheep and goats have similar isotope values that range between − 19.0‰ and − 20.0‰ for carbon and between 5.5 and 8.4‰ for nitrogen, reflecting the consumption of C_3_ plants. The five cattle samples have a relatively narrow δ^15^N value range of 2.4‰ (from 7.2 to 9.6‰), but a much larger δ^13^C value range of 11.4‰ (from − 18.5 to − 7.1‰), which indicates differential access to C_4_ plants.

## Discussion

### Cat diets in Qalhât and Siraf

Due to their peculiar ecological and behavioral features, cats can be both autonomous predators as well as dependent on food provisioning by humans^23,76^. Hunting of wild prey is more significant in rural areas, where anthropogenic food is scarcer^13^. Cats that are independent on food availability by humans and their activities, have been shown to exhibit low δ^13^C and δ^15^N values and narrow value ranges, which reflect a specialized trophic niche that is based on small wild animals^17,23^. Cats from urban contexts, however, are expected to exhibit a wide δ^13^C value range that reflects access to a variety of food sources through humans, as well as high δ^15^N values, especially if marine resources are available^17^.

The cats included in the present study come from urban contexts. In Siraf, cat remains were discovered in the commercial heart of the town, the area of the Great Mosque, which was surrounded by shops^36,47^, while in Qalhât, they were found in various buildings across the medieval town^43^. Thus, it is expected that, either by direct feeding and/or scavenging on human food scraps, the Siraf and Qalhât cats consumed foods that they would not be able to access otherwise, for example, meat from large mammals, such as sheep and cattle, as well as fish.

In Qalhât, cats have a 0.4‰ lower mean δ^13^C value, but a 2.8‰ higher mean δ^15^N value than the marine fish, which indicates that marine protein contributed significantly to the diet of the cats from this site. The fact that cats have a similar mean δ^13^C value to the finless porpoise and the small delphinid, but a 1.5–2.0‰ higher mean δ^15^N value, supports this interpretation and further indicates that cats consumed protein of higher trophic level than these two marine mammals, which commonly feed on small fish, crustaceans, cephalopods and other molluscs. Moreover, although cats and red foxes target the same prey groups^77^, the red fox from Qalhât has a 7.0‰ lower δ^15^N value than the mean δ^15^N value of the cats, reflecting the consumption of small prey that was of lesser dietary significance for the domestic cats. Seabirds as well as rats feeding on both fish and seabirds could have also been part of the diet of the Qalhât cats^78–81^. According to our FRUITS model, rats and birds (including seabirds) may have constituted, respectively, ~ 16% and ~ 21% of the dietary protein consumed by these cats.

Although the mean δ^13^C value of the Siraf cats is only 0.3‰ lower than the mean δ^13^C value of the cats from Qalhât, the 4.8‰ mean δ^15^N value difference between the cats from the two sites, suggests the consumption of lower trophic level foods by the Siraf cats. This mean value difference in nitrogen is statistically significant (p < 0.001). Having a 3.1‰ lower mean δ^13^C value and a 0.6‰ higher mean δ^15^N value compared to the analyzed fish, cats from Siraf were less dependent on marine protein than the cats from Qalhât. This is further supported by the results of the mixing model (Supplementary Table S6), which suggest that ~ 41% of the protein consumed by the cats from Qalhât derived from marine resources, compared to only ~ 34% for the cats from Siraf, with herbivorous mammals comprising their highest dietary contribution (~ 37%). Therefore, the high mean δ^13^C value of the cats from Siraf most likely indicates their dietary dependence on animals who fed on C_4_ plants, with a small contribution of protein from marine sources for those with higher δ^15^N values.

### Different cat diets and local baselines

Although both Siraf and Qalhât are coastal sites (Fig. 1) and were major harbors during the investigated periods^42,47^, cats from the two sites seem to have had different diets. For urban cats, anthropogenic food comprises an important part of their diet^13^. Hence, the different diets observed in the cats from the two sites may be related to the composition of waste resulting from human food preparation and consumption on the one hand and its accessibility and corresponding feeding opportunities offered in the cats’ respective home ranges on the other. Although it was not possible to analyze human bone samples from either site, archaeological and historical evidence provide information on the availability of food sources in Siraf and Qalhât during the studied periods.

Historical information and archaeobotanical evidence indicate that fruit (especially dates), cereals (including wheat, rice, sorghum and millet) and vegetables (e.g., garlic and eggplant) were either cultivated and/or imported in pre-Islamic and Islamic Oman^72,74,75,82^, including medieval Qalhât^73,83^. The three rat specimens with lower δ^15^N values that were primarily feeding on crops, corroborate the presence of such plants at the site and likely their importance in human diets. Archaeological evidence of plant remains, such as dates^84^, grapes^85^, millet and wheat^86,87^, is also present in prehistoric Iran. Crops, such as wheat and barley, were paid as taxes during the medieval period^88^, when water mills close to Siraf were likely used for grinding cereals^47^. According to the zooarchaeological record, subsistence in Qalhât and Siraf was based on marine fish and domestic animals (Table 1). Hunting of wild mammals and birds was rarely practiced and occasionally larger marine vertebrates (turtles and delphinoids) were captured, probably as a bycatch of fishing activities. The proportion of the domestic animal bones is higher in Siraf, suggesting that fish contributed less to the human diet in this site compared to Qalhât. Historical sources nevertheless support a high consumption of fish both in Oman^35^ as well as in the coast of the Arabian Gulf^34^. In Oman, both small fish, such as sardines and anchovies, as well as larger fish, such as sharks and sailfish, were consumed likely after being sun-dried or salted^35^. Thick layers of fish bones discovered in the domestic building B94, where some of the cat remains analyzed in the present study were also found, may indicate the use of the building for fish processing activities^89^. Such activities, however, may have also taken place on the beach, providing subsistence opportunities to animals, such as seabirds, rats and cats^35^.

In Qalhât, pelagic fishing seems to have been more important than coastal, inshore fishing (Table 1). The 18 pelagic and four coastal fish from this site have a lower mean δ^13^C value (− 11.3 ± 1.1‰ and − 12.1 ± 1.1‰, respectively) compared to the four pelagic (− 9.6 ± 0.9‰) and 16 coastal (− 8.9 ± 1.0‰) fish from Siraf (Fig. 2, Supplementary Table S3). In the Arabian Gulf, high temperatures and high evaporation rates lead to hypersaline conditions^90,91^, which result in the underdevelopment of the mangrove tree populations^92,93^. A benthic trophic pathway in microphytobenthos sites, which are ^13^C-enriched compared to mangrove sites^94,95^, may, thus, account for the higher δ^13^C values of the fish from Siraf. Moreover, fish from Qalhât, both coastal and pelagic (Fig. 2), have higher δ^15^N values compared to fish from Siraf (Fig. 3), which indicates perhaps a longer trophic chain in the Oman Gulf (although the δ^15^N value differences between fish from the two Gulfs could be also attributed to baseline δ^15^N variations^96^). The high trophic level of the fish available in Qalhât is seen in the high δ^15^N values of the cats that probably consumed fish, such as longtail tuna and requiem shark, which are the most represented fish categories from the site^37^, and/or seabirds and rats that fed on fish^78,80,81^.

The zooarchaeological evidence from both sites indicates also that among the domestic species, sheep and goats were the most important, with a representation in the skeletal assemblage of > 90% (Table 1). In Qalhât, the wide value ranges (δ^13^C: 1.7‰ and δ^15^N: 2.9‰) for sheep and goats suggest a diet with various amounts of C_4_ protein (perhaps partially at coastal areas and salty grounds) as well as potentially a dietary contribution of marine protein (especially for one sheep with a δ^15^N value of > 15.0‰). Similar isotopic signatures were observed in the ovicaprines from the Islamic site of Qalʿat al-Baḥrayn in Bahrain^97^. The practice of using small fish, especially sardines, as fodder has been reported for Oman by late thirteenth and fourteenth century CE sources^34,35^. Although slightly more present in Siraf (3.0%) than in Qalhât (0.6%), cattle were rare, probably due to the animal’s size that requires larger quantities of water to be sustained compared to sheep and goats^98,99^. Cattle are also less tolerant to drinking saline water^100,101^. Coastal Iran and Oman have an arid climate, with low annual precipitation, leading to freshwater shortages and increased salinity, while the majority of the freshwater used originates from groundwater resources^102,103^. Of the five cattle from Siraf, three have isotope values that indicate a high dependence on C_4_ plants. There is a high diversity of wild C_4_ plants in the region^104–106^, while archaeobotanical evidence of millet proves that it has been cultivated in the area for centuries before the studied period^86,87^. Although, at first glance, cattle could not have contributed significantly to the diet of the cats given their low overall representation to the faunal assemblage, it was noted that the Siraf cat remains have been collected in the debris deposited near the Great Mosque, more precisely in the commercial heart of the urban center characterized by shops, where butchers likely practiced their craft as well. Cattle butchers in particular not only need large markets with good sales opportunities, but also produce a lot of offal per capita. Therefore, the comparatively large number of cats in the excavation area in question as well as their isotopic signatures could possibly relate to the presence of such a guild. Additionally, considering that the site has been partially excavated^47^ and only 10% of the recovered faunal remains has been analyzed^36^, it is likely that large accumulations of cattle bones associated with butchery activities, such as those reported for early Byzantine Sagalassos in Turkey^107^, were not discovered. An alternative source of C_4_ protein for the cats from Siraf could have been small prey that fed on C_4_ plants. Yet, the two rats analyzed have lower δ^13^C and similar or higher δ^15^N values than the cats, while the consumption of mainly wild prey would likely result in lower δ^15^N values, similarly to the red fox from Qalhât. This brings us back to the previous point that economic activities in the city center opened up opportunities for Siraf cats to get hold of food with a high C_4_ content.

The zooarchaeological evidence from Siraf seems to suggest a lesser dependence on marine protein by humans in the area compared to Qalhât. This is further supported by the isotope values of animals such as dogs and chickens, which have previously been considered as proxies for human diets^97,108,109^ (however, see also Refs.^110,111^) and which have mean δ^13^C values indicative of a terrestrial diet. The higher δ^13^C values of the cats compared to these two animals (mean value difference of about 6.0‰) suggests that, although the marine dietary signal in the cats from Siraf is not as clear as for the cats from Qalhât, cats from both sites seem to represent a stronger proxy for the marine resources available in the two regions. This has also been suggested for the Islamic site of Qalʿat al-Baḥrayn, where isotopic analysis of both humans and fauna revealed that despite the large numbers of fish bones at the site, humans and chicken (likely consuming human food scraps) have isotope values that indicate a primarily terrestrial diet, while one cat has a higher δ^13^C value that indicates a higher dietary dependence on marine protein^97^.

A higher dependence on marine protein for cats, compared to animals that were perhaps mainly feeding on the waste of human food preparation and consumption, likely indicates that these felids were roaming freely, scavenging both on human food scraps as well as on refuse related to on-site processing of fish. The clearer isotopic signal of a marine diet for the cats from Qalhât probably relates to more extended fishing activities in this site, which further included large pelagic species. Finally, cats (and animals in general) are viewed favorably in Islamic culture^112,113^, however, the high mortality of juvenile cats in Qalhât along with the presence of skeletal pathologies (including traumatic lesions) observed in at least two cats^43,45^, may be partially indicative of occasional human violence towards cats. An overall lower human tolerance of cats in this town could have restricted them to the harbor, where large fish were probably gutted prior to further processing. Conversely, the cats that roamed the city center of Siraf may have frequented mainly the immediate surroundings of the market place with its shops, where the processing and sale of fish may have been one activity among many.

### Cat diets at seaport sites

**Figure 4:**
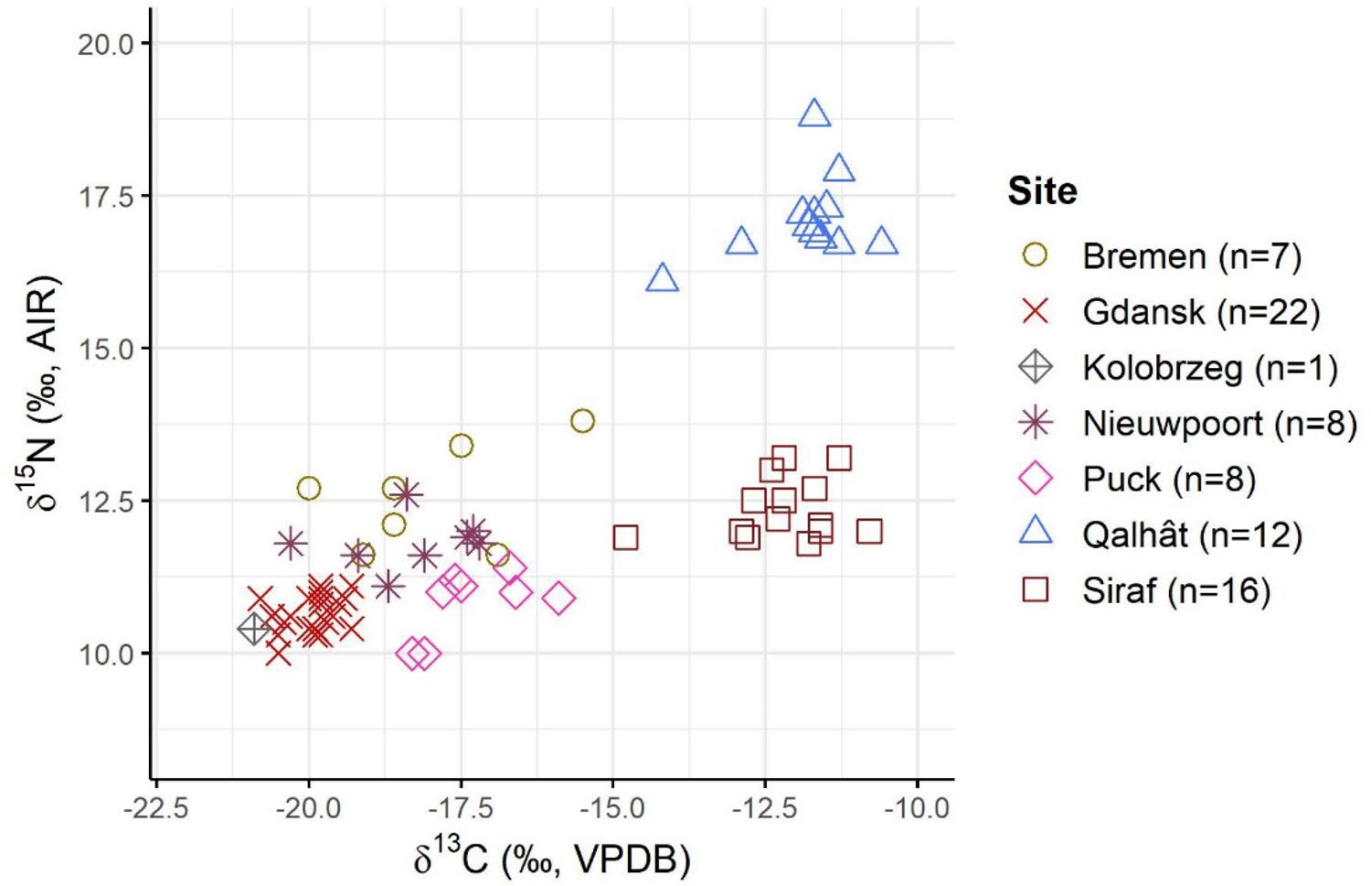
δ^13^C and δ^15^N values of cats from the medieval harbor sites of Qalhât (Oman) and Siraf (Iran) (present study) as well as from Bremen (Germany), Nieuwpoort (Belgium) and Puck, Gdańsk and Kołobrzeg (Poland)^17^.

A marine dietary signal has also been reported for cats from medieval port towns in Europe. In a recent study, Krajcarz et al.^17^ analysed cat remains from various sites, both coastal and inland, in Poland, Germany and Belgium and reported higher δ^13^C and δ^15^N values for the cats from the coastal sites. Krajcarz et al.^17^ further reported δ^13^C and δ^15^N differences between cats from port sites located in the Baltic Sea region (Puck, Gdańsk and Kołobrzeg in Poland) and those found in the North Sea region (Bremen in Germany and Nieuwpoort in Belgium), analogous to isotopic value differences reported for fish by Barrett et al.^114^, i.e., both δ^13^C and δ^15^N values being lower for the Baltic Sea, where there is a larger freshwater input. Although cats from Qalhât and Siraf have higher δ^13^C and δ^15^N values than the Polish, German and Belgian cats reported by Krajcarz et al.^17^ (Fig. 4, Supplementary Table S4), these differences are most likely related to local isotopic baselines. What remains evident, however, from both studies, is that cats are opportunistic animals that adjust their diets based on the availability of local resources.

## Conclusions

By establishing local isotopic baselines, this study generated new evidence on the diet of cats from the medieval harbor sites of Siraf in Iran and Qalhât in Oman. The δ^13^C and δ^15^N results suggest different dietary habits, with the cats from Qalhât being highly dependent on fish and animals feeding on fish, and the cats from Siraf having a mixed marine-terrestrial (C_4_) diet. This C_4_ signal is potentially related to the consumption of cattle meat and offal, however, additional isotopic analyses (i.e., hydrogen stable isotope analysis^115^ and compound-specific stable isotope analysis of amino acids^116,117^) may help to provide a clearer dietary reconstruction in the future.

With an augmented interest in keeping cats as pets^118^ and research showing the benefits for mental health in living with companion animals (e.g., Refs.^119,120^), it becomes increasingly important to learn more about the interactions between humans and cats in space and time. By reconstructing the diet of domestic cats from two harbors in the Arabian Gulf and the Gulf of Oman, this study revealed that these felids were most likely roaming freely, scavenging on both the waste of human food consumption as well as refuse related to fishing activities, and, thus, representing an enhanced proxy for the marine dietary protein component of the human diet. The dietary differences between cats from Siraf and Qalhât indicate either differences in the availability of marine resources and/or distinct living conditions, induced by the specific feeding opportunities offered in the anthropogenic space of the two sites. A dietary reconstruction of domestic cats from various sites and periods could shed more light on how humans and cats interacted, thereby creating both unique bonds and mutual dependencies^121,122^ that resulted in cats being one of the most popular pets in the world today^118^.

## Supplementary Information

Supplementary Information.

## Data Availability

All data generated or analyzed during this study are included in this published article (and its Supplementary Information file).
